# Elevated Oxytocin Levels and Their Relationship to Metabolic Syndrome and Obesity Among Young Sudanese Adults: A Cross-Sectional Analysis

**DOI:** 10.7759/cureus.81451

**Published:** 2025-03-30

**Authors:** Liena B Mekki, Yasir H Elhassan, Rehab M Badi, Ahmed F Shazali, Moaz A Mojaddidi, Sara Ibrahim, Hussein A Abdalla, Zeinab Elsawaf, Alfarazdeg A Mohammedali, Amal Saeed

**Affiliations:** 1 Department of Medical Physiology, Elrazi University, Khartoum, SDN; 2 Department of Basic Medical Sciences, Taibah University, Madinah, SAU; 3 Department of Physiology, King Khalid University, Abha, SAU; 4 Department of Physiology, Africa International University, Khartoum, SDN; 5 Department of Physiology, Faculty of Medicine, University of Khartoum, Khartoum, SDN

**Keywords:** insulin resistance, metabolic syndrome, obesity, oxytocin, sudanese population

## Abstract

Aims: This study was to determine the prevalence of metabolic syndrome (MetS) and obesity in young adults from Sudan, investigate their effect on plasma levels of oxytocin, and examine the association of oxytocin level with the determinants of metabolic syndrome and obesity.

Methods: This was a cross-sectional study that included 202 young adults. Random sampling was applied to recruit volunteers from Ribat University, Khartoum, Sudan. Blood pressure, waist circumference, and plasma lipids were measured and applied for the diagnosis of metabolic syndrome according to the National Cholesterol Education Program - Adult Treatment Panel III (NCEP ATP III) criteria. Fasting blood glucose and insulin were used to validate the insulin resistance in patients with MetS. Oxytocin was measured using enzyme-linked immunosorbent assay (ELISA).

Results: Metabolic syndrome was diagnosed in 29 students (14.36%). Mean oxytocin level was significantly higher in students with MetS (10.34 pg/mL, N=29) compared with students without MetS (9.15 pg/mL, N=174) (P < 0.001). Mean oxytocin level did not differ between students with homeostatic model assessment for insulin resistance (HOMA-IR) ≥ 2.5 (N=47), and without HOMA-IR < 2.5 (N=155); the oxytocin level was 9.53 pg/mL and 9.25 pg/mL, respectively (P = 0.068). Moderate positive correlations existed between oxytocin and waist circumference, BMI, body weight, triglycerides (TG), hip circumference, and high-density lipoprotein (HDL; P < 0.001). Oxytocin did not correlate with fasting blood glucose.

Conclusion: Oxytocin levels increased in young adults with MetS. Oxytocin levels correlate positively with BMI, waist circumference, blood pressure, triglycerides, and HDL.

## Introduction

Oxytocin, also known as the "love hormone," has garnered increased attention beyond its conventional roles, such as childbirth and lactation, due to its involvement in metabolic processes related to obesity and energy expenditure [1, 2]. Oxytocin is a cyclic nonapeptide hormone synthesized primarily in the paraventricular and supraoptic nuclei of the hypothalamus and secreted by the posterior pituitary gland [3,4]. Although it is widely recognized for its roles in childbirth and lactation, oxytocin also functions as a neuromodulator involved in social behavior, stress regulation, appetite control, and energy expenditure. Recently, research has increasingly focused on its potential role in metabolic processes such as obesity and energy balance [3, 4]. Recent work has highlighted the multiple functions of oxytocin in relation to food intake and energy balance. Research has shown that oxytocin plays a pivotal role in regulating appetite and energy metabolism. Studies have shown that administering oxytocin can significantly decrease caloric intake in humans and animal models, making it a potential target for therapeutics against obesity [1, 3, 4].

The hypothalamus, a brain region central to appetite regulation, is a key target of oxytocin. In addition to modulating neural pathways that govern hedonic and homeostatic feeding behaviors [3], oxytocin has been shown to enhance metabolic processes. For glucose uptake, oxytocin appears to stimulate the translocation of GLUT-4 transporters to the cell membrane in insulin-sensitive tissues such as skeletal muscle and adipose tissue, leading to increased glucose uptake [5]. Regarding lipid metabolism, oxytocin promotes lipolysis in adipose tissue and enhances lipid oxidation in skeletal muscle, thereby contributing to improved lipid utilization and reduced insulin resistance [5].

The association between oxytocin and MetS is particularly important when considering gender differences. While several researchers have demonstrated that hormonal fluctuations during the menstrual cycle affect oxytocin levels and their metabolic effects in females, emerging evidence also suggests that oxytocin plays a significant role in male physiology. In males, oxytocin has been implicated in regulating appetite, modulating stress responses and cardiovascular function, as well as maintaining metabolic homeostasis. Moreover, circulating oxytocin in men has been linked to favorable metabolic parameters, which may contribute to enhanced insulin sensitivity and improved energy balance. These findings underscore the need for further research into the sex-specific mechanisms by which oxytocin influences metabolic regulation [2, 6]. The previous information is essential for young adults in Sudan, as their understanding could provide insights into sex-specific interventions for MetS. For example, studies have shown that oxytocin may have sex-specific effects on appetite and energy expenditure, thus requiring individualized therapies [7,8].

Furthermore, it is noteworthy that oxytocin has significant implications for inflammation and adiposity. Preclinical studies suggest that oxytocin not only exerts lipolytic effects on white adipose tissue but may also promote the browning of white adipocytes into beige cells, thereby enhancing thermogenesis and energy expenditure through upregulation of thermogenic markers such as UCP1. In addition, oxytocin has been shown to exert anti-inflammatory actions by modulating cytokine production within adipose tissue, contributing to improved metabolic homeostasis [7,8].

Chronic inflammation within adipose tissue is one of the underlying causes of obesity and metabolic syndrome (MetS). Oxytocin has been shown to reduce inflammation in fat cells and improve insulin sensitivity, thereby potentially mitigating some complications associated with obesity [9]. However, oxytocin also acts on central nervous system regions involved in appetite regulation and energy balance, which may result in increased food intake and weight gain [7]. Thus, while oxytocin’s peripheral effects could be protective against metabolic derangements through the reduction of adipose tissue inflammation and improvement of insulin sensitivity, its central effects could conversely predispose individuals to MetS. These dual, context-dependent actions underscore the complexity of oxytocin’s role in metabolic regulation and highlight the need for further investigation to determine the conditions under which one effect predominates over the other.

Metabolic syndrome is defined as a cluster of interrelated risk factors for cardiovascular disease and type 2 diabetes. Although various organizations propose slightly differing definitions, the National Cholesterol Education Program-Adult Treatment Panel III (NCEP ATP III) criteria [10] are widely accepted.

One of the several causes of MetS is stress [11]. Chronic psychological stress has been proven to be associated with MetS [11]. Stress disturbs the hormonal balance of the hypothalamic-pituitary-adrenal axis, causing an elevated blood cortisol level, which leads to high glucose and insulin levels [12]. In turn, insulin-mediated effects on adipose tissue promote visceral adiposity, insulin resistance, dyslipidemia, and hypertension [13].

The prevalence of MetS is increasing worldwide. In Sudan, the prevalence of MetS was 16.6% among young adults using the ATP III definition of MetS and was detected only in obese as opposed to underweight, normal weight, and overweight individuals [14].

We conclude that there is increasing evidence that oxytocin interacts complexly with MetS, particularly among young people [1, 2]. Oxytocin's capacity to control appetite, increase energy utilization, and decrease inflammation in adipose tissues makes it an attractive target for developing treatments against obesity-related diseases [3,7]. We should conduct more research to uncover these associations in diverse populations like the Sudanese and devise strategies to combat the global MetS epidemic.

In this study, we aimed to discover if there is any correlation between oxytocin and MetS in Sudanese young adults. We chose young adults because development is almost complete, sexual hormones and gender differences have already developed, and the effect of aging and environment is less than in the older adult group.

This study evaluated the association between plasma oxytocin levels and MetS (diagnosed using the NCEP ATP III criteria) and obesity (determined by BMI) in young adults, alongside homeostatic model assessment for insulin resistance (HOMA-IR) as an additional measure of insulin resistance, which is closely tied to metabolic disturbances.

The specific objectives of this study are to compare the mean plasma levels of oxytocin in students diagnosed with MetS with those in students without the syndrome and to evaluate the correlation between plasma oxytocin levels and the various components of MetS, namely waist circumference, fasting blood glucose, systolic and diastolic blood pressure, high-density lipoprotein cholesterol (HDL-C) levels, and triglyceride levels.

Based on previous literature suggesting that oxytocin modulates both central appetite regulation and peripheral metabolic processes, our overarching hypothesis is that elevated oxytocin levels are significantly associated with the presence and severity of metabolic syndrome and its key components in Sudanese youth, and that this association may differ between males and females.

## Materials and methods

This cross-sectional study was conducted among young Sudanese adults at National Ribat University in Khartoum, Sudan, from August 1, 2021, to July 31, 2023. The study aimed to determine the prevalence of MetS and obesity and to investigate the relationship between oxytocin levels and the components of MetS and obesity. National Ribat University is a private institution; thus, its student body primarily represents a high socioeconomic class. Although individual socioeconomic data were not collected, this inference is based on the university’s profile and admission trends, which indicate that students predominantly come from high-income backgrounds. This characteristic minimizes potential confounding factors such as low-income-induced dietary deficiencies and stress, which are known to negatively influence metabolic health. Consequently, the associations observed between oxytocin levels and components of MetS are more likely to reflect underlying physiological processes rather than socioeconomic disparities.

The 16- to 25-year-old age group represents a population that has completed growth and full development of one's hormones and is also far from the onset of menopause. The selected age group (16-25 years) was chosen to minimize the variability in hormonal levels due to developmental or aging-related factors. However, we acknowledge that other factors, including stress, may still influence hormonal profiles among individuals.

Relevant to MetS, this specific subset aims to aid in understanding the condition by using lower age thresholds and possibly targeting young adults at the primary level for the prevention of MetS. This group's focus is to investigate the potential impact of oxytocin on metabolic processes, which can help develop individualized treatment approaches for individuals with MetS.

Inclusion criteria

All male and female undergraduate students aged between 16 and 25 years from National Ribat University who were in good general health and willing to provide informed written consent were eligible for inclusion.

Exclusion criteria

Students were excluded if they had any serious illnesses (e.g., tuberculosis, renal failure), chronic endocrine or metabolic disorders if female students were pregnant or lactating, or if they were taking any medications known to affect metabolic parameters (such as corticosteroids like prednisone and dexamethasone, which have well-documented effects on glucose metabolism, weight gain, and fat distribution [12,13]; thyroid medications such as levothyroxine, which directly affect the metabolic rate; antidiabetic agents including insulin, metformin, and sulfonylureas, which alter blood glucose and insulin dynamics; and lipid-lowering drugs, for example, statins such as atorvastatin and simvastatin, known to modify lipid profiles [10]).

The study included randomly recruited male and female students from Ribat University, Khartoum, Sudan. Random sampling was chosen instead of convenience or systematic sampling to enhance representativeness, reduce selection bias, and facilitate valid statistical inferences. Each person had the same opportunity to be selected; thus, the sample population reflected the diversity of the population in terms of various factors like age, gender, or socioeconomic status. The implementation involved selecting participants by a random number generator to enhance the randomness and scientific validity of the study outcomes.

The number of students enrolled was based on a calculated sample size using the formula (N = Z² x P x Q/ E2 ), whereas N: sample size, Z: factor (1.96), P: prevalence (0.166), Q: 1-P (0.837), and E: error allowed (correction factor = 0.05 for 95% confidence level) ((1.96)² x0.166 x 0.837/(0.052) = 214). [15]

Initially, the study included 250 students. However, the final number of samples included in the analysis was 202 because 28 were withdrawn due to incomplete data collection (16 were withdrawn due to damage to the blood sample, and four were excluded after data entry as they were frank outliers).

The following information was obtained for each volunteer: gender, age, past medical history, family history (hypertension, angina pectoris, diabetes mellitus, and hyperlipidemia), and the level of physical activity per week; demographic data (weight, height, hip circumference, and waist circumference); and blood pressure. Fasting blood samples were collected after a 12-hour fasting period and used for the following measurements: fasting plasma glucose, fasting lipid profile (total cholesterol (TC), HDL-C, low-density lipoprotein cholesterol (LDL-C), triglycerides (TG)), fasting insulin, and oxytocin levels.

Handling of blood samples

Blood samples were collected in ethylenediaminetetraacetic acid (EDTA)-coated tubes and processed within 30 minutes of collection. Samples were centrifuged at 1,500 g for 10 minutes at 4°C, and the obtained plasma was aliquoted and stored at -80°C until assay. To preserve oxytocin stability, samples were subjected to a single freeze-thaw cycle prior to analysis.

Calculation of HOMA-IR

Fasting blood glucose was measured from blood samples using an enzymatic glucose oxidase assay on an automated analyzer (Hitachi 902; Roche Diagnostics, Mannheim, Germany). Calculation of HOMA-IR was done using the standard formula, with conversion factors adjusted appropriately for the sample type.

Assessment of diet and physical activity

Dietary intake was assessed using a validated food frequency questionnaire, and physical activity levels were quantified using the International Physical Activity Questionnaire (IPAQ) to provide objective and reproducible measures. These variables were included as covariates in the statistical models.

Menstrual cycle considerations

Although data on the menstrual cycle were not collected in this study, we acknowledge that phase-specific hormonal variations may influence circulating oxytocin levels. 

Serum levels of oxytocin

Oxytocin was measured using a commercial enzyme-linked immunosorbent assay (ELISA) kit (Cayman Chemical, Michigan, IL, USA) with 9.6% intra-assay and 6.3% inter-assay coefficients of variation reported at a 46.9 pg/mL concentration. Serum and plasma (EDTA as the anticoagulant) samples were extracted using solid-phase extraction (C18-E 200 mg/3 mL columns, Phenomenex, Torrance, CA, USA) to eliminate interfering molecules and to concentrate the sample for analysis following the provider's instructions.

Serum insulin

Serum insulin was measured using the sandwich enzyme immunoassay technique, ELISA. Quantitative measurement of insulin in human serum was conducted using ELISA kits (ST AIA-PACK IRI) from Tosoh India Private Limited, Bhiwandi, India. The kits provide a detection range of 0.5 - 320 µU/mL.

Diagnosis of MetS, obesity, and insulin resistance

Metabolic syndrome was diagnosed using NCEP ATP III criteria [10]. According to these criteria, participants were classified as having MetS if they presented with any three or more of the following conditions: abdominal obesity (waist circumference greater than 102 cm in males or 88 cm in females), hypertriglyceridemia (TG levels equal to or exceeding 150 mg/dL), low HDL-C levels (lower than 40 mg/dL in males or lower than 50 mg/dL in females), and hypertension (systolic/diastolic blood pressure equal to or exceeding 130/85 mmHg or treatment with antihypertensive medication). We utilized the World Health Organization’s international BMI cut-off points for classifying individuals. Participants with a BMI ≥25 kg/m² were classified as overweight, while those with a BMI ≥30 kg/m² were considered obese [14]. Height and weight were measured using standardized protocols, and BMI was calculated as weight (kg) divided by the square of height (m). Insulin resistance was defined as HOMA-IR ≥ 2.5. HOMA-IR was calculated using the equation HOMA-IR = (glucose in mmol/L x insulin in mIU/mL)/22.5 [16].

Data analysis and statistics

Data entry and analysis were performed using IBM SPSS Statistics software, version 20 (IBM Corp., Armonk, NY, USA). Categorical variables were compared using the Chi-square test or Fisher's exact test. The normality of continuous data was evaluated using the one-sample Kolmogorov-Smirnov test. Since not all variables followed the normal distribution except fasting blood glucose, the non-parametric Mann-Whitney U test was used to compare the means of the two groups. To investigate the interrelationship between oxytocin and the different components of MetS and obesity, we applied Kendall's tau-b correlation analysis. Statistical significance was considered at a p-value ≤ 0.05.

Ethical considerations

The ethical approval committee in the Faculty of Graduate Studies and Scientific Research at the National Ribat University approved the study (approval number: Ph.D/Phy/2020/no:1). The research aims and sampling method were described to the randomly selected students. Informed written consent was obtained from those who agreed to participate, which clearly stated the freedom to withdraw from the study at any stage and assured the privacy of the data collected.

## Results

Correlation between oxytocin and MetS

Oxytocin increased significantly with metabolic syndrome; mean oxytocin was 9.15 pg/ml in students without MetS and 10.3 pg/ml in students with MetS, P=0.000, as shown in Table 1.

**Table 1:** Demographic and clinical characteristics of students with or without metabolic syndrome (MetS) Statistical significance was calculated using the Mann-Whitney U test; statistically significant (p-value < 0.05); effect of sex: none of the significant differences was affected by sex. BMI: body mass index; WC: waist circumference; HC: hip circumference; WHR: waist to hip ratio; SBP: systolic blood pressure; DBP: diastolic blood pressure; TC: total cholesterol; TGS: triglycerides; HDL-C: high-density lipoprotein cholesterol; LDL-C: low-density lipoprotein cholesterol; HOMA-IR: homeostatic model assessment for insulin resistance.

Parameters	Students without MetS				Students with MetS				Total students				P-value
	N=173 (94 male, 79 female)				N=29 (15 male, 14 female)				N=202 (109 male, 93 female)				
	Min	Max	Mean	SD	Min	Max	Mean	SD	Min	Max	Mean	SD	
Age (years)	16	25	19.1	1.8	16	25	20.3	2.5	16.0	25.0	19.3	2.0	0.019
BMI (kg/m^2)^	15.26	49	24.49	5.49	20.31	51.21	32.45	7.15	15.26	51.21	25.6	6.4	0.000
WC (cm)	56	156	83.3	14	77	123	102.4	13.1	56.0	156.0	86.0	15.4	0.000
HC (cm)	73.0	140.0	101.3	12.63	83.0	150.0	115.4	15.30	73	150.0	103.0	13.9	0.000
WHR	0.63	1.34	0.82	0.08	0.75	1.26	0.89	0.1	0.63	1.34	0.8	0.1	0.000
SBP (mmHg)	100	150	114.2	10.1	110	150	127.8	10.2	100.0	150.0	116.2	11.1	0.000
DBP(mmHg)	60	100	77	8.9	60	110	89.3	11.2	60.0	110.0	78.7	10.2	0.000
TC (mg/dl)	90	416	156.9	34.5	111	336	178.2	45.5	90.0	416.0	160.0	36.9	0.007
TGS (mg/dl)	85.9	253.6	132.3	22.2	110	260.5	164.2	33.5	85.9	260.5	136.9	26.5	0.000
HDL-C (mg/dl)	25.6	75.5	41.6	7.3	32.8	86.8	51.8	11.6	25.6	86.8	43.1	8.8	0.000
LDL-C (mg/dl)	25.2	289.8	88.9	28	40.6	197.1	93.5	32	25.2	289.8	89.5	28.5	0.638
Glucose (mg/dl)	48	130	92.5	13.2	68	137	105.7	18.2	48.0	137.0	94.4	14.7	0.000
Insulin (µU/ml)	0.3	273.1	14.2	40	0.8	255.8	41.7	68.1	0.3	273.1	18.2	45.9	0.000
HOMA-IR	0.04	59.54	3.35	8.91	0.21	59.94	11.14	17.64	0.04	59.94	4.5	10.9	0.000
Oxytocin pg/ml	7.76	12.92	9.15	0.78	8.86	13.22	10.3	1.06	7.76	13.22	9.3	0.9	0.000

Prevalence of MetS in the study population

Data analysis included the results obtained from 202 students: 93 females and 109 males. The age of the study population ranged between 16 and 25 years. Metabolic syndrome was diagnosed in 29 students (14.4%), which included 14 female and 15 male students; hence, 15.1% of female students were diagnosed with MetS compared to 13.8% of male students (P = 0.794; Table 1).

This provides summary statistics for the measurements performed in students with (N=29) or without the MetS (N=173). When comparing male and female students separately, the patterns of significant variation between students with MetS and students without MetS from the same sex did not change.

Prevalence of MetS among different weight categories

Amongst the 45 obese students, 20 (44.4%) suffered from MetS. On the contrary, only 5.73% (9/149) of non-obese individuals were diagnosed with MetS (P=0.000). Consequently, obese young adults were more than 10 times as likely to suffer from MetS as their non-obese counterparts (odds ratio 13.16%, 95% CI = 5.383 to 32.151). The odds of suffering from MetS were 16.9 times higher in obese young males compared to non-obese individuals, as illustrated in Table 2. 

**Table 2:** The significant effect of sex on the relationship between obesity and metabolic syndrome (MetS), with a higher odd ratio in males. P-values were calculated using Pearson’s chi-square test (Fisher’s exact test was applied when expected frequencies were <5). A p-value <0.05 was considered statistically significant.

Metabolic syndrome (MetS)		Obesity								
		Females			Male			total		
		Not obese	Obese	Total	Not obese	Obese	Total	Not obese	Obese	Total
No MetS	Count	72	7	79	76	18	94	148	25	173
MetS	Count	6	8	14	3	12	15	9	20	29
Total	Count	78	15	93	79	30	109	157	45	202
Odds ratio, P-value		13.714, P=0.000			16.89, P=0.000			13.16, P=0.000		
											95% Confidence interval		3.69- 50.95			4.311- 66.165			5.383 - 32.151		

Oxytocin levels in young Sudanese adults

The mean oxytocin level in young Sudanese adults included in the study was 9.32 ± 0.913 pg/ml, ranging from 7.76 pg/ml to 13.22 pg/ml. Oxytocin levels were slightly but significantly lower in young adult females compared to males (P=0.044; Table 3).

**Table 3:** Oxytocin level in young Sudanese adults and the effect of sex and body weight on mean oxytocin level * Compared to normal weight; 1 compared to obesity Class 1; 2 compared to obesity Class 2; 3 compared to obesity Class 3 Significant variation between obesity classes 1 and 2 persisted in males (P=0.000, N=21 and 7 ) and females (P=0.040, N=7 and 2); significant variation between obesity classes 1 and 3 persisted in males (P=0.029, N=21 and 2) and females (P=0.003, N=7 and 6); significant variation between classes 2 and 3 was lost in males (P=0.078, N=7 and 2) and females (P=0.046, N=2 and 6).

Oxytocin with gender and BMI		Oxytocin (pg/ml)								
		Count	Min	Max	Mean	Standard deviation	Standard error of mean	75 percentile	95 percentile	P-values
Young Sudanese adults		202	7.76	13.22	9.32	0.91	0.06	9.65	11.10	-
Gender	Male	109	7.78	12.92	9.40	0.91	0.09	9.71	11.05	0.044
	Female	93	7.76	13.22	9.22	0.92	0.10	9.50	11.39	
BMI Kg/m^2^	Underweight	19	7.76	8.89	8.27	0.32	0.07	8.50	8.89	0.000*
	Normal weight	84	7.85	10.01	9.04	0.47	0.05	9.43	9.71	-
	Overweight	54	7.84	9.68	9.05	0.36	0.05	9.34	9.62	0.887*
	Obese	45	9.48	13.22	10.59	0.93	0.14	11.05	12.31	0.000*
Underweight	Male	11	7.78	8.89	8.32	0.30	0.09	8.46	8.89	0.657
	Female	8	7.76	8.69	8.21	0.36	0.13	8.54	8.69	
Normal weight	Male	41	8.17	10.00	9.04	0.46	0.07	9.39	9.68	0.925
	Female	43	7.85	10.01	9.04	0.48	0.07	9.47	9.73	
Overweight	Male	27	7.84	9.68	9.17	0.35	0.07	9.37	9.67	0.002
	Female	27	8.50	9.59	8.93	0.32	0.06	9.18	9.55	
Obese	Male	30	9.62	12.92	10.48	0.86	0.16	10.85	12.31	0.379
	Female	15	9.48	13.22	10.79	1.05	0.27	11.45	13.22	
Obesity categories	Obesity class 1	28	9.48	12.31	10.06	0.54	0.10	10.18	10.71	0.000*^,2,3^
	Obesity class 2	9	10.69	12.16	11.01	0.46	0.15	11.05	12.16	0.000*^,1^ 0.002 ^3^
	Obesity class 3	8	11.25	13.22	11.96	0.73	0.26	12.45	13.22	0.000 *^,1^ 0.002 ^2^

Being underweight significantly reduced oxytocin levels, and obesity significantly increased oxytocin levels (P=0.000 for both).

Mean oxytocin levels were significantly affected by variations in BMI. The mean level of oxytocin in normal-weight young adults was 9.04 ± 0.47 (SD) pg/ml and ranged from 7.9 to 10 pg/ml. Table 3 shows that the mean oxytocin level increased with increased weight category, being of significantly lower mean levels in underweight individuals (8.27 ± 0.32 pg/ml) and rising to maximum mean levels (11.96 ± 0.73) in individuals with morbid obesity (obesity Class 3). The mean level of oxytocin in normal-weight adults was similar in males and females; hence, sex variations in oxytocin level depended on BMI category.

The relation between oxytocin levels and age, different measurements of obesity, and components of MetS

The strength of the relationship between oxytocin and different possible predictors was evaluated by calculating Kendall's tau_b correlation coefficient (Τ, tau) (Table 4).

**Table 4:** Correlation of oxytocin with age, body measurements, and the different components of metabolic syndrome, showing that the strongest association was with waist circumference. The strength of association was calculated using Kendall's tau_b correlation coefficient. (*=P-value<0.05; **= P-value < 0.001). HC: hip circumference; WC: waist circumference; BMI: body mass index; SBP: systolic blood pressure; DBP: diastolic blood pressure; TGs: triglycerides; HDL-C: high-density lipoprotein cholesterol; LDL-C: low density lipoprotein cholesterol; HOMA-IR: homeostatic model assessment for insulin resistance

Parameter correlation to oxytocin	Correlation coefficient			Strength of association for total population
	Males (N=109)	Females (N=93)	Total (N=202)	
Age (year)	0.042	0.056	0.058	No association
Weight (Kg)	0.607^**^	0.384^**^	0.515**	Moderate
HC (cm)	0.507^**^	0.328^**^	0.444**	Moderate
WC (cm)	0.640^**^	0.475^**^	0.581**	Moderate
BMI (kg/m^2)^	0.680^**^	0.452^**^	0.572**	Moderate
SBP (mmHg)	0.234^**^	0.179^*^	0.245**	Weak
DBP (mmHg)	0.176^*^	0.254^**^	0.235**	Weak
Glucose (mg/dl)	0.059	0.074	0.057	No association
Cholesterol (mg/dl)	0.091	0.118	0.073	No association
TGs (mg/dl)	0.525^**^	0.376^**^	0.452**	Moderate
HDL-C (mg/dl)	0.527^**^	0.376^**^	0.391**	Weak
LDL-C (mg/dl)	-0.135	-0.065	-0.122*	Very weak
Insulin (µU/ml)	0.197^**^	0.128	0.161**	Very weak
HOMA-IR2	0.183^**^	0.132	0.153**	Very weak

**Figure 1:**
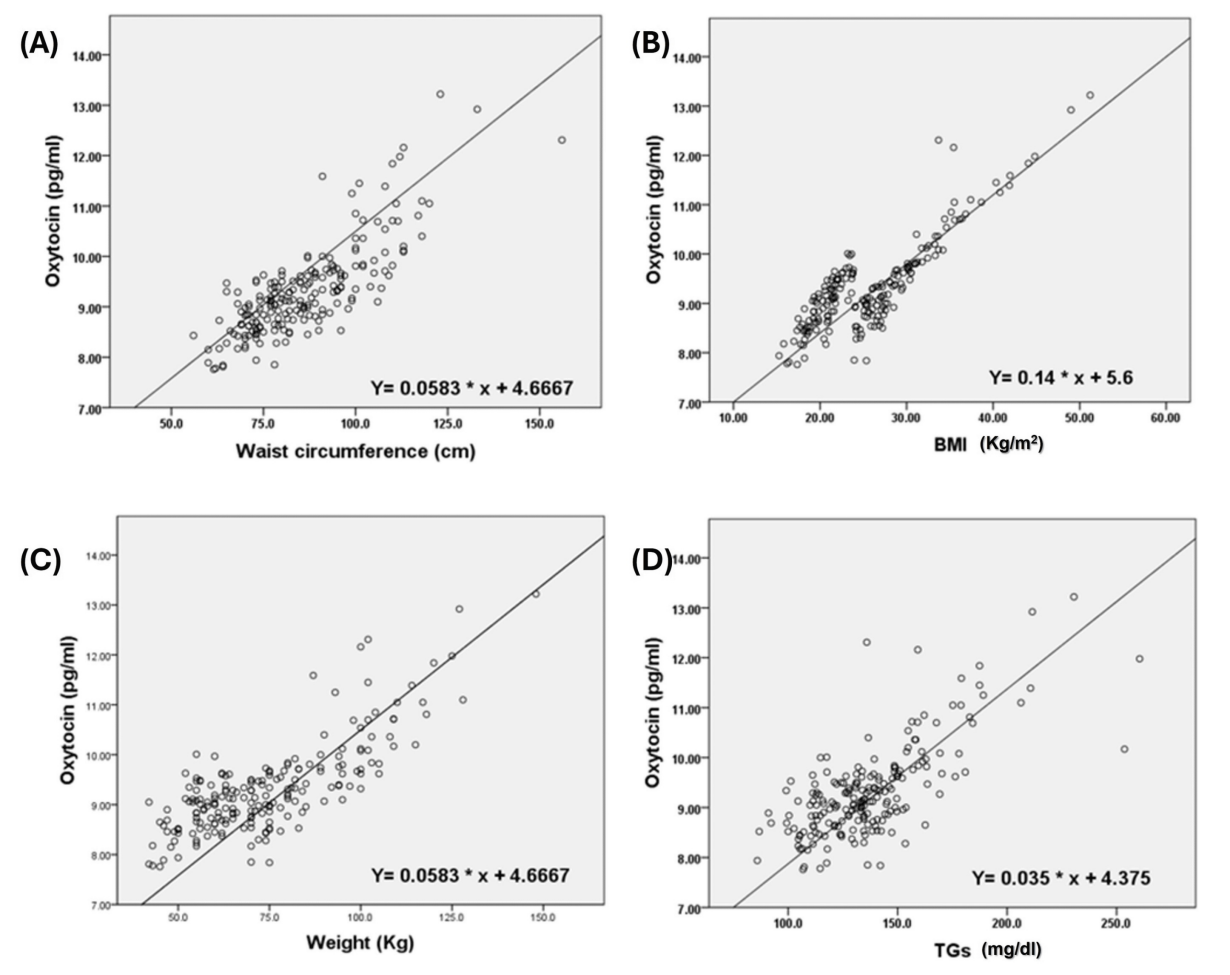
Scatter plots illustrate the moderate positive correlations between plasma oxytocin levels and waist circumference, BMI, body weight, and triglyceride (TG) level. Each panel represents individual data points with an overlaid trendline depicting the direction and strength of the correlation. The graphs visually support the statistically significant moderate positive associations identified in Table 4. Axes are labeled with the corresponding measurement units.

Regarding the total population, the strongest association was the moderate association detected between oxytocin and waist circumference (T= 0.581, P=0.000). followed by the BMI (T = 0.572, P = 0.000), body weight (T = 0.515, P = 0.000), triglyceride level (T = 0.452, P = 0.000), and hip circumference (T = 0.44, P = 0.000). The strength of the association between the investigated parameters and oxytocin was greater in males than in females, except for diastolic blood pressure, and the association was stronger in females than in males. The relationships between oxytocin and waist circumference, BMI, weight, and triglycerides are also shown in four different graph plots, further enhancing the previous statistical analysis (Figure 1).

## Discussion

Oxytocin

Our findings that oxytocin levels are elevated in students with higher BMI and larger waist circumference are in line with previous studies indicating that oxytocin is involved in appetite regulation, fat storage, and energy balance [1,3,4,7]. In several animal models, oxytocin administration has been shown to reduce food intake while also influencing fat distribution [5,7]. This pattern suggests that the observed rise in oxytocin, particularly in individuals with increased visceral adiposity, could be interpreted as a compensatory response aimed at counteracting metabolic dysfunction.

However, it is important to note that not all individuals with obesity develop MetS, as evidenced by the well-recognized distinction between metabolically healthy and unhealthy obesity [14]. In our study, although oxytocin was strongly correlated with obesity-related measures such as BMI and waist circumference, its association with hyperglycemia was not significant. This discrepancy indicates that while elevated oxytocin levels may reflect the body’s response to increasing adiposity, this relationship does not necessarily extend uniformly to all criteria of MetS. In other words, the compensatory rise in oxytocin may be more closely linked to adiposity itself rather than to the full spectrum of metabolic abnormalities required for a diagnosis of MetS.

Taken together, these observations suggest the need for further research to elucidate the intricate relationship between oxytocin and the various components of MetS. Future studies should evaluate whether oxytocin elevation is a uniform biomarker for MetS or if it is predominantly a marker of obesity that becomes dysregulated only in certain metabolic contexts [1,17].

Mean serum oxytocin in students with MetS was significantly higher compared with students without MetS (10.34 pg/ml and 9.15 pg/ml, respectively, P<0.0001); our findings were supported by Szulc et al.'s cross-sectional study [17], which was conducted in 540 volunteer men, whose ages were between 50 and 85 years old. In contrast, in Al-Rawashdeh et al.'s case-control study [18], they found a negative correlation between oxytocin and metabolic syndrome in a study including Caucasian individuals, two-thirds females and one-third males, with an older age group (mean age 51 years old) and more obese (70.5 % of the study population, mean BMI = 33 kg/m²). This difference in an association can be justified by the difference in ethnicity, age group, and obesity characterizing the majority of the population. It can also be due to the more significant percentage of their study population being females, as the female gender appeared to have a less strong association between most of the metabolic syndrome criteria parameters and oxytocin, as shown in Table 4. In Guoyue's study [19], among 170 subjects, 75 with MetS and 95 non-MetS reported that in middle-aged Chinese individuals, lower levels of oxytocin were associated with MetS. Surprisingly, individuals diagnosed with MetS in Guoyue's study [19] had a mean BMI of 26.76 kg/m², which is less than in our study (32.45); 20/29 of students with metabolic syndrome were obese (having a BMI > 30 kg/m²) in our study; this is about 68.97% of students diagnosed with MetS; consequently, this suggests that both ethnic group and BMI affect the correlation of oxytocin and metabolic syndrome.

Oxytocin was also significantly correlated to components of MetS criteria, further underscoring its potential role in metabolic regulation [20, 21]. In our study, oxytocin correlated with waist circumference, BMI, HDL-C, TG, and blood pressure. Lawson's review article [1] and several other studies supported these findings.

Oxytocin may act primarily on hypothalamic pathways that regulate appetite and fat storage rather than directly affecting pancreatic beta (β)-cells or insulin signaling. Several studies have suggested that oxytocin improves lipid oxidation but has minimal impact on glucose uptake in muscle and liver tissues [1,3,5]. This mechanistic insight supports the idea that the observed surge in oxytocin in subjects with higher BMI and waist circumference may represent a compensatory response aimed at maintaining energy homeostasis and mitigating further metabolic dysfunction.

It is important to note that while the correlations between oxytocin and individual MetS criteria are modest when examined separately, the cumulative pattern, encompassing obesity measures, lipid profiles, and blood pressure, suggests a broader regulatory role for oxytocin. Individually, the associations may not appear robust; however, the integrated effect across multiple parameters supports the hypothesis that even minor changes in oxytocin levels may contribute to systemic metabolic homeostasis. This perspective is further reinforced by previous findings [1, 17, 19] that indicate a nuanced relationship between oxytocin and metabolic dysfunction. Nonetheless, we agree that further investigations are needed to establish whether oxytocin could serve as a comprehensive biomarker for MetS.

The significant positive correlation between oxytocin and BMI and waist circumference was reinforced by Szulc et al.'s cross-sectional study [17] in men between 50 and 58 years old and Weingarten et al.'s cohort study [22] in Germany, which included men and women aged 47.7 +/- 15.2 years old. Moreover, Schorr et al. [23] found a correlation between oxytocin and BMI in 59 women aged between 18 and 45. In contrast, Binay et al. [24] found that oxytocin was significantly lower in obese children with a mean age of 14 years; it was even lower with MetS and had a negative correlation with waist-hip ratio, but this can be due to the difference in age range between the two studies. This may indicate that the correlation between oxytocin and BMI may vary according to the age group between children and adults. More studies need to be done to investigate the relationship between oxytocin and BMI in children and adults.

Oxytocin increased significantly with HDL-C, as stated in Fu-Man et al.'s study [25] in China. Oxytocin significantly increased with HDL-C in 151 obese and 160 non-obese women of childbearing age. Lawson et al. [4] showed a correlation between oxytocin and HDL. Besides, the positive correlation between oxytocin and TG was supported by Weingarten et al. [22].

Oxytocin level was correlated with high blood pressure and positively correlated with systolic and diastolic blood pressure; this correlation was supported by Weingarten et al. [22]. In a previous study, regular hugs between partners were associated with lower blood pressure and higher oxytocin levels in premenopausal women [26]. In an animal experimental study, oxytocin also decreased arterial blood pressure [27]. Besides, in a human interventional study, intranasal oxytocin significantly reduced blood pressure [28]. The previous information might indicate (that oxytocin is one of the body's homeostatic mechanisms used to control the increase in blood pressure) that high oxytocin, found cross-sectionally in hypertensive students or students with higher blood pressure, is an internal process to curb the surge in blood pressure, as oxytocin has shown to have this potential ability in previous studies [27, 28].

In the total population, mean oxytocin was higher in the male gender (9.40 pg/ml) than in the female gender (9.22 pg/ml; P=0.044), but in normal-weight individuals, mean oxytocin was 9.04 pg/ml for both males and females. In the study by Al-Rawashdeh et al. [18], oxytocin was found more in males than females. Furthermore, this study provides reference values for oxytocin levels in young adult Sudanese individuals (Table 3); as not much research has been conducted regarding oxytocin in Sudan, these values can be used as reference values for evaluating the levels of oxytocin in individuals of similar age and ethnic groups.

Metabolic syndrome and obesity

Metabolic syndrome among young adults in Sudan should be a considerable issue; in this study, the prevalence of MetS was 14.4%, and though the study population was within the single age group (young adults aged between 16 and 25), the mean age of students with MetS was significantly higher than those without, which agrees with previous evidence of increased risk of MetS with age [17]. This high prevalence will lead to an even higher increase in the risk of ischemic heart diseases in the following years in Sudan; this is a tendency that has been detected in various populations globally [29].

This high prevalence is supported by Yasir et al.'s previous study [14], where the prevalence of MetS was found to be 16.6% using the same criteria in young Sudanese adults.

Metabolic syndrome was significantly associated with obesity, which affected 22.3% of the study population. Obesity increased the risk of MetS by more than 10-fold; 44.4% of obese students suffered from MetS, whereas only 5.73% of non-obese students were diagnosed with MetS (P=0.000).

Other studies in which similar tendencies across diverse populations have been documented dependably show a correlation between obesity and MetS [30]. This strong correlation is explained by the fact that obesity is a precursor to various metabolic dysfunctions, including dyslipidemia and hypertension [29].

Recommendations, strengths, and limitations of the study

Further research into the oxytocin-BMI foundation across different ages and racial variability parameters appears to be a worthy path to pursue to untangle the understanding and practical application of the variables set. There is a possibility that oxytocin concentrations may be an indicator in screening for people who are likely to develop MetS, particularly in regions where obesity and metabolic diseases are rampant.

The strengths of this study are the wide-scope analysis, where the paper describes in detail the variance that exists regarding metabolic and health measures concentration of oxytocin within a specific population group, and the use of a particular ethnic group, which provides information on the reference values of oxytocin concentrations in the blood of young Sudanese males and females, which is lacking in the current literature.

This study is limited by its cross-sectional design, as its observational nature means that the relationship between oxytocin concentrations and metabolic disease cannot be conclusively established. It is also limited by the age group restriction, which prevents it from examining how oxytocin affects various metabolic parameters throughout life. The small sample size also limits the study; studies undertaken with small sample sizes are likely to produce different or even incongruous results because of the anomaly of random variation. In turn, larger sample sizes decrease the impact of this randomness, resulting in more accurate conclusions. Moreover, the population specificity impacts the findings' generalization to other ethnic groups or populations.

Another limitation of our study is the lack of detailed menstrual cycle phase data for female participants. As the menstrual cycle can modulate circulating oxytocin levels, this factor may have contributed to inter-individual variability. Future studies should incorporate menstrual cycle tracking to address this potential confounder more comprehensively.

## Conclusions

In conclusion, the findings from this research are essential in understanding oxytocin levels in Sudanese youth in relation to various metabolic and health parameters. Notably, higher oxytocin levels were significantly associated with BMI and with select key components of MetS, specifically, increased waist circumference, elevated blood pressure, lower HDL-C, and higher TG levels, rather than with all indicators uniformly. Additionally, our study found no significant differences in oxytocin levels between genders, although levels were nonsignificantly higher in males. These results highlight the complex and nuanced role of oxytocin in metabolic regulation and underscore the need for further investigation into its potential as a biomarker in diverse populations.
